# Prevalence and determination of species distribution of *Eimeria* in poultry from the Swabi district, Pakistan

**DOI:** 10.14202/vetworld.2024.1983-1989

**Published:** 2024-09-01

**Authors:** Noreen Begum, Sumaira Shams, Farhad Badshah, Irfan Khattak, Muhammad Salman Khan, Naimat Ullah Khan, Warda Naz, Eliana Ibáñez-Arancibia, Patricio R. De los Ríos-Escalante, Seema Hassan, Mourad Ben Said

**Affiliations:** 1 Department of Zoology, Abdul Wali Khan University, Mardan, Pakistan; 2 State Key Laboratory of Animal Biotech Breeding, Institute of Animal Science, Chinese Academy of Agricultural Science, Beijing 100193, China; 3 Shenzhen Branch, Guangdong Laboratory of Lingnan Modern Agriculture, Key Laboratory of Livestock and Poultry Multi-Omics of MARA, Agricultural Genomics Institute at Shenzhen, Chinese Academy of Agricultural Sciences, Shenzhen 518000, China; 4 College of Veterinary Sciences and Animal Husbandry, Abdul Wali Khan University, Mardan, Pakistan; 5 Department of Zoology, Hazara University, Mansehra, Pakistan; 6 PhD Program in Sciences Mentioning Applied Molecular and Cell Biology, La Frontera University, Temuco, Chile; 7 Department of Chemical Engineering, Laboratory of Engineering, Biotechnology and Applied Biochemistry, Faculty of Engineering and Science, La Frontera University, Temuco, Chile; 8 Department of Biological and Chemical Sciences, Faculty of Natural Resources, Catholic University of Temuco, Temuco, Chile; 9 Department of Nucleus of Environmental Sciences, Faculty of Natural Resources, Catholic University of Temuco, Temuco, Chile; 10 Department of Basic Sciences, Higher Institute of Biotechnology of Sidi Thabet, University of Manouba, Manouba 2010, Tunisia; 11 Laboratory of Microbiology, National School of Veterinary Medicine of Sidi Thabet, University of Manouba, Manouba 2010, Tunisia

**Keywords:** coccidiosis, domestic chickens, *Eimeria* species, microscopic and molecular identification, Pakistan, prevalence

## Abstract

**Background and Aim:**

Coccidiosis, caused by protozoan parasites of the genus *Eimeria*, is a significant concern in poultry farming, leading to substantial economic losses worldwide. In Pakistan, poultry is a major component of the agricultural sector, with both broiler and egg-laying chickens playing crucial roles in meeting the country’s protein needs. Despite the importance of the poultry industry, there is limited data on prevalence and species distribution of *Eimeria* in different types of chickens in District Swabi, Khyber Pakhtunkhwa, Pakistan. This study aims to estimate the prevalence and determine the distribution of *Eimeria* species in broiler and egg-laying chickens in this region.

**Materials and Methods:**

Nine hundred fecal samples were collected from broiler (380) and egg-laying domestic chickens (520) in District Swabi, Pakistan. Microscopic analysis was used to identify *Eimeria* parasites in all samples. After microscopic examination for positive identification, *Eimeria* species were determined using polymerase chain reaction (PCR) assays.

**Results:**

Microscopic examination identified *Eimeria* oocysts in 44.4% (400/900) of the samples. *Eimeria* parasite infection significantly varied based on chicken type, age, and gender (p < 0.05). The study found that broiler chickens (52.63%, 235/450), young chickens (4–6 weeks) (55.5%, 285/500), and females (52.2%, 200/380) were more infected with *Eimeria* spp. than egg-laying domestic chickens (38.5%, 200/520), adults (above 6 weeks) (28.8%), and males (36.7%, 165/450). PCR indicated a distribution rate of 42.5% (170/400) *Eimeria tenella*, 26.25% (105/400) *Eimeria acervulina*, 20% (80/400) *Eimeria maxima*, and 11.25% (45/400) *Eimeria mitis*. None of *Eimeria necatrix*, *Eimeria brunetti*, or *Eimeria praecox* was found in the study.

**Conclusion:**

This study underlines the essential requirement for targeted interventions due to the prevalence and predominance of *E. tenella* among identified *Eimeria* species. Future research should focus on refined sampling strategies and investigate the clinical significance of these parasites for effective disease management in the local poultry industry.

## Introduction

The poultry industry significantly contributes to job generation and provides a vital animal protein source [1]. Asian countries have surpassed the growth rates of Australia and New Zealand in global chicken production. Notably, China, the US, Brazil, Russia, Mexico, India, and Pakistan have emerged as key players in the global chicken production market recently [2].

In Pakistan, the poultry sector is a robust contributor to the national economy, constituting 2.3% of GDP [3]. In addition, it significantly influences the stability of beef and mutton prices [4]. Poultry coccidiosis, a damaging bird disease prevalent worldwide, poses a significant challenge to the poultry industry. The financial impact of this ailment is significant due to increased mortality, reduced weight gain, and heightened necrotic enteritis susceptibility [5].

*Eimeria* oocysts, first identified in chickens’ ceca in 1891 [6], reveal the seriousness of the matter in light of historical context. Poultry coccidiosis, caused by species within the genus *Eimeria* of the phylum *Apicomplexa* and family *Eimeriidae*, is an enteric parasitic disease. This is the most common and economically damaging intestinal parasite disease for chickens worldwide [7]. Approximately 1800 species in the *Eimeria* genus inhabit the intestinal mucosa of diverse mammals and birds, underscoring its prevalent threat as a parasite [5].

*Eimeria* species, recognized for their monoxenous nature, depend solely on a single host for their life cycle due to their host specificity [8]. The seven coccidian species, including *Eimeria acervulina*, *Eimeria brunetti*, *Eimeria maxima*, *Eimeria tenella*, *Eimeria necatrix*, *Eimeria praecox*, and *Eimeria mitis*, exhibit varying degrees of pathogenicity. Among Enterococci species, *E. necatrix* and *E. tenella* are the most pathogenic, whereas the lesser pathogens include *E. acervulina*, *E. brunetti*, and *E. maxima*. In contrast, *E. mitis* and *E. praecox* are generally considered less pathogenic [9].

Each *Eimeria* species occupies a unique location within the intestine, highlighting their specialized parasitic behavior. The genus’s direct life cycle involves both sexual and asexual multiplication phases, encompassing gametogony (sexual reproduction and gamete creation), schizogony (agamogony/merogony), and sporogony formation [10]. Effective control measures are crucial, as most transmission happens through the fecal-oral route.

The global coccidiosis challenge is addressed through the application of anticoccidial drugs as a widespread control method [11]. The diagnosis of coccidiosis relies on various factors, including clinical features, host gut pathology, parasite morphology at different stages of parasitism, and the pre-patent period [12]. Identification of *Eimeria* species can be laborious and demands specialized knowledge. To address these limitations, recent advancements have introduced polymerase chain reaction (PCR) techniques, which offer a more efficient and precise means of identifying the *Eimeria* species infecting chickens [9].

Avian coccidiosis is a globally prevalent protozoan disease impacting poultry production, with a prevalence of 5% to 70% [2]. It is common in developing nations like Egypt and Pakistan. Pakistan’s poultry sector ranks as the second-largest in the country, supplying both broiler meat and eggs from domesticated chickens. This sector, producing 0.767 million tons of broiler meat and 12.857 million eggs yearly, contributes 4.8% to the agriculture sector’s value addition.

Despite the poultry industry’s significance, data on the prevalence and determination of species distribution of *Eimeria* in various types of chickens in Pakistan, especially in the Swabi district of Khyber Pakhtunkhwa (KPK), remains limited. Understanding the epidemiology of *Eimeria* infections is vital for implementing effective control measures to reduce the burden of coccidiosis. This study aimed to estimate the prevalence and distribution of *Eimeria* species in broiler and egg-laying chickens within District Swabi, Pakistan. Through the use of both microscopic examination and PCR assays, the study aims to estimate the dominant *Eimeria* species and analyze their relationship with factors such as chicken type, age, and gender.

## Materials and Methods

### Ethical approval and informed consent

This study was approved by Ethics Committee of Abdul Wali Khan University, Mardan, Pakistan (Approval no. AWKUM//Zoo/2021/3965). This approval ensured that all procedures related to the humane and ethical treatment of poultry during sample collection. The study was conducted with a commitment to animal welfare, and all measures were taken to minimize any potential distress or harm to the birds involved. Ethical considerations were addressed by obtaining verbal informed consent from all participants, ensuring their voluntary participation and confidentiality of their responses.

### Study period and location

The study was conducted from July 2021 to July 2022. The study area is District Swabi in Pakistan’s KPK province, which is part of the Mardan Division. It is located between the Indus and Kabul rivers. Before becoming a district in 1988, Swabi was a tehsil within the Mardan District. Swabi’s district is currently divided into four Tehsils: Swabi, Topi, Lahore, and Razar.

### Study design and sampling method

This study employed a cross-sectional analysis to estimate coccidiosis prevalence in broiler and egg-laying domestic chickens in District Swabi. Based on the previously reported prevalence rate of *Eimeria* infection in broiler chickens in Pakistan, which is 65% reported by Bachaya *et al*. [13], the sample size was calculated using the following formula:

n = [Z^2^ x p x (1-p)]/e^2^

Where n is the sample size, Z is the critical value of the standard normal distribution for a 95% confidence level (1.96), p is the estimated prevalence rate (65% = 0.65), and e is the desired margin of error (5% = 0.05).

Applying this formula, the minimal sample size required for our study, considering the 65% prevalence rate, was 350 samples from each of broiler and egg-laying chickens, resulting in a minimum total of 700 samples. Consequently, nine hundred fecal samples were collected and analyzed from broilers (380) and egg-laying chickens (520) in District Swabi, Pakistan.

### Sample and data collection

The fecal samples (n = 900) were evenly collected across the four Tehsils of District Swabi. Each Tehsil contributed 225 samples, encompassing 380 from broilers and 520 from domestic egg-laying chickens. This balanced allocation captured the varied dynamics of coccidiosis prevalence within distinct poultry populations. Supplementary information vital to the study was acquired through a questionnaire. The structured questionnaire focused on key parameters, including breed, gender, and age of the sampled avian subjects. These additional data unveiled potential correlations between coccidiosis prevalence and demographic factors.

### Morphological identification of Eimeria oocysts from fecal samples

Following the collection of fecal samples, the Laboratory of Epidemiology and Public Health (College of Veterinary Sciences and Animal Husbandry, AWKUM) analyzed the presence of *Eimeria* species. Morphological identification was achieved using the fecal flotation technique. In this method, a saturated solution of sodium chloride was used. Subsequently, the filtrate from samples was centrifuged to isolate the oocysts. The centrifugation process, conducted at 400× *g* for 10 min, facilitated the settling of the oocysts. The supernatant was then carefully discarded, and the *Eimeria* oocysts in the sediment underwent two rounds of washing with distilled water at 2500× *g* for 10 min each. This meticulous process ensured *Eimeria*’s purification of oocysts for accurate morphological identification under microscopic examination.

### Molecular identification of the Eimeria species

To accurately identify *Eimeria* species, genomic DNA was extracted from positive samples using the PureLink™ Microbiome DNA Purification Kit (Thermo Fisher Scientific, USA).

Subsequently, the extracted genomic DNA from all samples was subjected to a PCR assay. This PCR reaction was performed in a thermal cycler (Kyratec SC300G-R2 Australia), using species-specific primers designed for all seven *Eimeria* species. Refer to the table below for the specific primers, it employed in the amplification of genomic DNA (Table-1) [14, 15].

**Table-1:** Species-specific primers used in PCR for the detection of each *Eimeria* species.

*Eimeria* specie	Primer name	Primer sequences (5’- 3’)	Amplicon size (bp)	Annealing temperature (°C)	References
*Eimeria acervulina*	Ac-01F	AGTCAGCCACACAATAATGGCAAACATG	811	62	[14]
	Ac-01R	AGTCAGCCACAGCGAAAGACGTATGTG			
*Eimeria brunetti*	Br-01F	TGGTCGCAGAACCTACAGGGCTGT	626	65	[14]
	Br01R	TGGTCGCAGACGTATATTAGGGGTCTG			
*Eimeria tenella*	Tn-01F	CCGCCCAAACCAGGTGTCACG	539	62	[14]
	Tn-01R	CCGCCCAAACATGCAAGATGGC			
*Eimeria praecox*	Pr-01F	AGTCAGCCACCACCAAATAGAACCTTGG	354	60	[14]
	Pr-01R	GCCTGCTTACTACAAACTTGCAAGCCCT			
*Eimeria mitis*	Mt-01F	TATTTCCTGTCGTCGTCTCGC	327	58	[15]
	Mt-01R	GTATGCAAGAGAGAATCGGGA			
*Eimeria maxima*	Mx-01F	GGGTAACGCCAACTGCCGGGTATG	272	60	[14]
	Mx-01R	AGCAAACCGTAAAGGCCGAAGTCCTAGA			
*Eimeria necatrix*	Nc-01F	TTCATTTCGCTTAACAATATTTGGCCTCA	200	58	[14]
	Nc-01R	ACAACGCCTCATAACCCCAAGAAATTTTG			

PCR=Polymerase chain reaction

The PCR reactions were conducted in a total volume of 25 μL for each reaction, comprising 12. 5 μL mastermix (Thermo Scientific DreamTaq Green PCR Mastermix, Thermo Fisher Scientific), which included 3 μL genomic DNA, 1.5 μL each of reverse and forward primers, and 6.5 μL nuclease-free water. Standard cycle parameters were as follows: 1× (5 min at 94°C), 30× (1 min at 94°C, 90 s at 57°C, and 2 min at 72°C), and 1× (7 min at 72°C). After PCR amplification, the products were separated using 1.25% agarose gel electrophoresis, providing a visual representation of the amplified genes of *Eimeria* species. This approach allowed the precise identification and differentiation of *Eimeria* species based on their distinct primers (Table-1) [14, 15].

### Statistical analysis

The data were imported into Microsoft Excel 2019 (Microsoft Office, Redmond, WA, USA) and subsequently analyzed using Statistical Package for the Social Sciences version 18 software (IBM Corp., NY, USA). Coccidiosis prevalence was computed across various chicken groups, considering factors such as age, gender, month, and area. In addition, the prevalence of distinct *Eimeria* species was calculated. The Chi-square test was employed to scrutinize prevalence, with statistical significance set at p < 0.05.

## Results

### Microscopic examination

**Figure-1:**
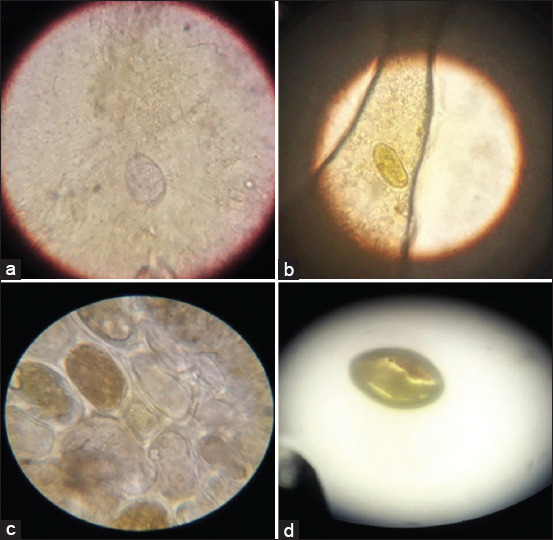
Microscopic observation images of *Eimeria* parasites (a: *Eimeria* *tenella*, b: *Eimeria maxima*, c: *Eimeria acervuline*, and d: *Eimeria mitis*).

Microscopic examination of fecal samples revealed *Eimeria* oocysts in 44.5% (400/900) of the investigated samples. The examination revealed various *Eimeria* oocysts, identified as oval and round bodies, under the microscope on prepared slides, as illustrated in Figure-1.

The prevalence of infection varied significantly according to several risk factors. In particular, broiler chickens exhibited a significantly higher infection rate (52.6%, 200/380) than egg-laying domestic chickens (38.5%, 200/520, p = 0.009). Young chickens (4–6 weeks old) had the highest infection rate among all age groups, with 55.5% (285/500) of samples tested positive. This was significantly higher than the prevalence in adult chickens (28.8% (115/400), p = 0.000). Finally, female chickens harbored *Eimeria* oocysts more frequently than males, with infection rates of 52.2% (235/450) and 36.7% (165/450), respectively (p = 0.003) (Table-2).

**Table-2:** Prevalence rates of *Eimeria* parasites, revealed by microscopy, in overall and according to risk factors.

Risk factors	Classes	Total number	Positive samples	Prevalence rates (% ± C.I.^1^)	p-value	Khi2
Category	Egg-laying domestic chickens	520	200	38.5±0.043	0.009*	6.779
	Broiler chickens	380	200	52.6±0.051		
Age	Young (4–6 weeks)	500	285	55.5±0.043	0.000*	28.514
	Adults (above 6 weeks)	400	115	28.8±0.045		
Gender	Male	450	165	36.7±0.045	0.003*	8.505
	Female	450	235	52.2±0.047		
Total		900	400	44.4±0.033		

1 :C.I.: 95% confidence interval,

* Statistically significant, p *<* 0.05

### Molecular identification

PCR amplification, performed on the 400 microscopically positive samples, confirmed the presence of four distinct *Eimeria* species in District Swabi (Figure-1). *E. tenella* emerged as the predominant culprit, infecting a staggering 42.5% (170/400) of the examined samples. Following closely were *E. acervulina* (26.3%, 105/400) and *E. maxima* (20%, 80/400), while *E. mitis* constituted a smaller portion (11.3%, 45/400). Notably, no evidence of *E. necatrix*, *E. brunetti*, or *E. praecox* was detected (Table-3).

**Table-3:** Distribution rates of each *Eimeria* species detected by molecular method performed on the 400 samples positive to the genus by microscopic examination.

*Eimeria* species	Positive sample	Distribution rate (%)
*Eimeria tenella*	170	42.5
*Eimeria acervulina*	105	26.3
*Eimeria maxima*	80	20.0
*Eimeria mitis*	45	11.3
*Eimeria brunetti*	0	0
*Eimeria necatrix*	0	0
*Eimeria praecox*	0	0
*Eimeria* spp.	400	100

## Discussion

The prevalence of coccidiosis in District Swabi, Pakistan, is in line with several reports on poultry health and parasitology from India [16], Pakistan [17, 18], Iraq [19], and Saudi Arabia [20]. In this study, the microscopic examination of 900 fecal samples yielded compelling results, revealing a substantial prevalence of *Eimeria* oocysts at 44.4%. This prevalence rate falls within the range observed in similar studies conducted in Nigeria [21] and Pakistan [22], highlighting the persistent challenge of coccidiosis in poultry farming. Our findings include records from diverse geographical regions, demonstrating variations in coccidiosis prevalence influenced by factors such as climate, management practices, and regional epidemiology. Our prevalence rate estimated at the District Swabi aligns with broader trends observed in South Asian poultry farming, where *Eimeria* infections continue to pose a significant threat to flock health [21].

Furthermore, our results highlight the multifactorial nature of coccidiosis prevalence, and the identification of chicken type, age, and gender as influencing factors in the District Swabi study is consistent with this understanding. Broiler chickens, young chickens (4–6 weeks), and females demonstrated higher susceptibility, a pattern observed across various poultry farming environments. In particular, younger chickens consistently exhibit increased vulnerability to *Eimeria* infections due to developing immune systems. The predisposition of broiler chickens to higher infection rates is a common finding, underscoring the importance of tailored management practices for specific poultry types. The heightened susceptibility of females aligns with the study of Oljira *et al*. [23] on gender-related variations in disease susceptibility among poultry, reflecting hormonal and physiological differences.

Moreover, the conformity of these findings with global trends enhances the robustness of the District Swabi study. This alignment emphasizes the universality of certain risk factors that influence *Eimeria* infection, transcending geographical boundaries. Integrating these results into our extensive database will contribute to a more nuanced understanding of the multifaceted factors shaping coccidiosis prevalence, fostering a comprehensive approach to poultry health management. By drawing parallels with our findings, the District Swabi study not only reinforces existing knowledge reported by Bachaya *et al*. [13] but also contributes region-specific insights. This information is invaluable for tailoring interventions to the unique dynamics of poultry farming in District Swabi, thereby enhancing the effectiveness of local disease management strategies.

The application of PCR assays to confirm the presence of various *Eimeria* species in District Swabi provides a molecular dimension to our understanding, aligning seamlessly with the advanced techniques present in our database on Parasitology. The distribution rates uncovered for *E. tenella*, *E. acervulina*, *E. maxima*, and *E. mitis* in the District Swabi study are consistent with global patterns observed in similar investigations. In particular, numerous studies globally have reported the prevalence of *E. tenella* in poultry populations [18]. For instance, a study conducted by Bachaya *et al*. [18] found *E. tenella* to be a prevalent species, particularly associated with severe clinical manifestations of coccidiosis. This aligns with the District Swabi study, which emphasizes the ubiquity of *E. tenella* and its potential impact on poultry health and productivity. In addition, Ahad *et al*. [24] provide insights into the distribution of *E. acervulina* in the Kashmir valley from February to January for 1 year. Their findings, similar to those of the District Swabi study, indicate that *E. acervulina* is a commonly identified species in poultry. Furthermore, Ahad *et al*. [24] highlighted the association between *E. acervulina* and milder clinical symptoms, contributing to a comprehensive understanding of the species’ impact on poultry production. A study conducted by Ahad *et al*. [24] has underscored the prevalence of *E. maxima* in poultry populations in the Kashmir Valley. Our identification of *E. maxima* aligns with the findings of Ahad *et al*. [24] emphasizing the widespread distribution of these species. Ahad *et al*. [24] also reported variations in pathogenicity among different *Eimeria* species, providing a valuable context for understanding the potential consequences of *E. maxima* infections. However, the presence of *E. mitis* in our study corresponds to studies such as that of Ahad *et al*. [24]. These researchers investigated the prevalence of *Eimeria* species in various regions of the Kashmir Valley, highlighting the diverse distribution of *E. mitis*. Their work contributes to the global understanding of *E. mitis* as a component of coccidiosis, providing context for the District Swabi study’s identification of these particular species.

The absence of *E. necatrix*, *E. brunetti*, and *E. praecox* in the positive samples, as determined by PCR, corresponds with the sporadic occurrences reported by Ahad *et al*. [24] and Ayaz *et al*. [25]. These reports also indicated that the prevalence of certain *Eimeria* species can exhibit regional variability. Molecular techniques employed in the present study not only enhance the accuracy of species identification but also contribute valuable data on the distribution and prevalence of specific *Eimeria* species within the local poultry population [24]. Ayaz *et al*. [25] have highlighted the global distribution of *Eimeria* species, emphasizing variations influenced by factors such as climate, management practices, and host characteristics. Our study enriches this understanding by providing region-specific insights into the prevalence of *Eimeria* species. The use of advanced molecular tools aligns with the contemporary trends in parasitology research, ensuring a more detailed characterization of the *Eimeria* landscape in the local poultry industry.

The dominance of *E. tenella* among the identified *Eimeria* species in District Swabi has critical implications for poultry health. Ahad *et al*. [24] emphasized that *E. tenella* is often associated with severe clinical manifestations of coccidiosis, including significant economic losses in affected flocks. The prevalence of this species in District Swabi underscores the urgency of targeted interventions to reduce the impact of coccidiosis on the local poultry industry. The proactive approach advocated by our study aligns with the best practices recommended in our database, emphasizing the importance of preventive measures to curb the spread of coccidiosis [20]. Tailored interventions, considering the prevalent *Eimeria* species, are crucial for reducing the economic burden on the poultry industry and safeguarding flock health [26]. Integrating these findings into our collective knowledge base facilitates a more targeted and context-specific approach to coccidiosis management, ensuring sustainable practices for local poultry stakeholders.

This study paves the way for future research in the management of coccidiosis. Our findings emphasize the ongoing importance of developing and fine-tuning approaches to counteract poultry parasitism. Focusing on refined sampling strategies is in line with the developing methodologies detailed by Haug *et al*. [27]. Sophisticated sampling methods, including molecular and spatial analyses, effectively uncover intricate epidemiological patterns. By adopting advanced sampling methods and examining complex health and economic consequences of *Eimeria* parasites, future studies can refine and enhance poultry management techniques, both locally for District Swabi and globally against coccidiosis.

## Conclusion

Our study in District Swabi, Pakistan, offers unique insights into the prevalence of coccidiosis and the distribution of *Eimeria* species among the local poultry population. With the use of microscopic examinations and molecular techniques such as PCR assays, the intricacies of coccidiosis in the region have been further elucidated. The significance of specific *Eimeria* species, their prevalence, and the associated economic and health consequences emphasize the need for targeted strategies in poultry agriculture for long-term success. Our findings, recognized for their limitations and potential biases, expand the current knowledge base and pave the way for upcoming investigations. To improve the comprehension of coccidiosis trends, develop innovative disease management tactics, and boost the well-being of the poultry sector in District Swabi, suggestions for fine-tuned sampling techniques and supplementary diagnostic approaches are suggested.

## Authors’ Contributions

NB and SS: Conceptualization and methodology. WN, NUK, SH, FB, and MSK: Software and validation. NB and IK: Formal analysis. NB and IK: Investigation, writing–original draft preparation, and data analysis. WN and MBS: Data curation. IK, MBS, and PDLRE: Writing-review and editing and data analysis. EIE and MBS: visualization. NB, SS, and IK: Project administration. All authors have read and agreed to the published version of the manuscript.
